# Building resilience of street-connected children during COVID-19: turning rights into practices through CINI method

**DOI:** 10.3389/fpubh.2024.1353867

**Published:** 2024-07-17

**Authors:** Debapriya Bhattacharyya, Swati Chakraborty

**Affiliations:** Child In Need Institute, Kolkata, India

**Keywords:** street children, child rights, health rights, SDG, COVID-19, resilience, street-connected children, participatory research

## Abstract

This article examines the methods and opportunities for SCC’s meaningful participation that recognize their agency and are aligned with General Comment No. 21 (GC21) to the United Nations Convention on the Rights of the Child (UNCRC) on Children in Street Situations (UNCRC, 1989). This article explains the application of CINI’s core practice models which explains the child centrality in development practices for “turning rights into practice for children” derived from the *Sustainable Development Goals, principles of Human Rights, UNCRC, and General Comment No. 20 (2016). The Institutional knowledge was practiced through child-led action research with street- connected children which resulted in the* development of agency among SCC, peer researchers, and child advocates for resilience building within their community during COVID-19. Child in Need Institute (CINI) has been working with SCC since 1989 and has derived a rich body of experience from the interventions. CINI applied participatory approaches to practice, research, decision-making, and policy development; thus, facilitating children in the process of systematically gathering information with their peers, identifying key issues and problems faced by SCC, and securing support from duty bearers that were required for the survival within their situations. Drawing on the approach undertaken and the tools used in the participatory research and advocacy, this article reflects upon the processes and strategies that worked out in facilitating SCC’s ability to exercise agency and resilience through evidence generation and advocacy during COVID-19 and the associated lockdown and beyond. Through capacity building on research tools, leadership and communication skills, SCC can build concrete evidence of their vulnerabilities and the gaps that pose as barriers to their access to existing support mechanisms. This evidence helps them to prioritize the solutions that are required to bring changes in their lives and that of their peers, with which they can advocate at different platforms that promote dialogs and negotiations between children and duty-bearers. A participatory research project funded by Wellcome Trust focused on the vulnerabilities faced by street-connected young people and the access to services available to them. It revealed the lack of understanding regarding SCC and their invisibility in data and planning of support services, the gaps in access to healthcare services, the social determinants of health including safety, and their exclusion in platforms for dialogs with duty bearers. They took these issues to local government leaders, service providers, and national and international advocacy platforms; and suggested solutions to local and world leaders to bring changes in their situations. This resulted in a marked increase in the responsiveness of service providers toward SCC during the period of COVID-19, and the increased agency and negotiation skills of peer leaders to support their communities and demand solutions during the period of COVID-19 and associated lockdown.

## 1 Introduction

SCC are acknowledged to be a part of the urban phenomenon, as mentioned by the Subgroup report on Child Protection for 11th 5 year Plan by the Working Group on Child Rights (Ministry of Women and Child Development, 2011). With rapid urbanization and rising urban population, the Asia Pacific region is home to the highest number of megacities globally, with China and India leading the region with the highest number of megacities (very large cities with more than 10 million people). Kolkata, situated in the eastern part of India, is one of the three mega cities in the country where the Child in Need Institute (CINI) has had its operations since 1989. CINI, with its human rights-based integrated approach of facilitation and service delivery, is driven by the CINI method, the conceptual framework that guides the entire CINI’s operation, from long and short-term strategic planning and programming to organizational management. CINI has been adopting a human rights- based approach along with child centrality as its core approach. The CINI Method is designed to translate child rights principles into practice (1). The CINI Method includes a three-tier framework for promoting a rights-based approach:

- Building Child-Friendly Communities (CFC) to bridge the last mile in the implementation of children’s rights at the field implementation level
- Building Child-Friendly Systems responsible for fulfilling children’s rights at the community and higher levels.
- Building Child-Friendly Organizations to equip organizational structures and management systems in adopting rights-based approaches in programs and operations.

The first, critical level in the model, the Child-Friendly Communities (CFC) approach, seeks to bring rights closer to children, by implementing The CINI Method at the critical level of the local community. CFC follows 7 building blocks which have undergone participatory design, field testing, implementation, and progressive corrections for over a decade. CFC is not a program but an approach. It provides a step-by-step methodology to influence processes for children from a rights perspective. It is a means to translate children’s rights into development practice by the institutions that are duty-bound to be accountable for the child. The principles that are followed while applying the method include participation, especially of children, setting accountability of duty-bearers, bringing multi-sectoral convergence and prevention.

CINI’s intervention with SCC, in the context of participation and inclusion, plays a very crucial role because it focuses on resilience by bringing about innovative and inclusive ways of equipping children as defenders of their rights. It incorporates participatory processes and methodological approaches toward the inclusion of SCC in planning, programs, policies, and decisions.

## 2 Method

In January 2019, CINI and StreetInvest (now CSC) began a participatory research project funded by Wellcome Trust focused on the vulnerabilities faced by street-connected young people and the services available to them (2). The project was designed to reflect Article 24 (Health and Health Services) and General Comment No. 21 (on Children in Street Situations) of the United Nations Convention on the Rights of the Child and Sustainable Development Goal No. 3 (Good Health and Well-being). The project also aimed to meet the demands for improved data for marginalized populations that underlie these, by providing a substantial evidence base that can: (1) inform alternative policies relating to SCC’s rights, health and well-being; and (2) inform the design of programs interventions which support and promote SCC’s rights, health and well-being. The research approach applied a child rights lens, using the framework of the UN Convention on the Rights of the Child (1989) to which India is a signatory, and the UN General Comment 21 (3) on the rights of children in street situations, authoritative guidance for states on how to meet their obligations to realize the rights of SCC.

The following methodology was followed in the participatory action research:

1. City-wide web-based mapping of spots with the highest concentration of SCC and their numbers.
2. Cross-verification of locations with High-Risk Area list prepared by WHO, Kolkata.
3. Selection of five jurisdictions with the highest concentration of SCC within the city.
4. Democratic election of peer leaders from SCC’s groups in the five jurisdictions.
5. Identification of 450 peers by peer leaders through snowball sampling, distributed uniformly in the five city jurisdictions.
6. Capacity building of street-connected peer leaders on research tools, leadership and communication.
7. Facilitation of street-connected peer leaders in the development of research tools.
8. Child-led testing of research tools and incorporating required changes on the basis of their feedback.
9. Quantitative data collection by peer leaders through Rapid Situation Assessment Surveys among 450 street-connected peers.
10. Peer-led focus group discussions among 60 SCC.
11. In-depth interviews with 22 service providers including healthcare service providers, police, local elected representatives, etc.
12. Analysis of findings involving peer leaders through consultations.
13. Dissemination of findings to SCC, communities, and duty bearers through a variety of resources such as comics, animations, and briefing papers.
14. Development of recommendations to share with service providers and policymakers by peer leaders.
15. Child-led prioritization of vulnerabilities revealed through research.
16. Child-led identification of duty bearers for advocacy.
17. Advocacy visits by peer leaders to duty bearers for sharing of vulnerabilities and joint discussions about solutions.
18. Support mechanisms ensured sanitation, food security and shelter during COVID-19through child-led mobilization of resources from local elected representatives, health centers and police stations.
19. Peer health education on Sexual and Reproductive Health Rights by peer leaders to 450 street- connected children.
20. Child-led advocacy by peer leaders in local, national and international platforms such as the United Nations Commission on Human Rights, United Nations Office on Violence Against Children and the United Nations Committee on Economic, Social and Cultural Rights (CESCR).
21. Reflection on vulnerabilities faced by SCC in international policies and guidelines such as the United Nations General Comment 26.
22. Acknowledgement of peer leaders’ actions by the United Nations Special Representative to the Secretary-General on Violence Against Children.

### 2.1 Participants

Thirty ‘Street Champions,’ SCC and young people aged 13–18, were trained on evidence collection tools, directly conducting research activities from data collection to analysis and the development of recommendations. Initially, team members from CINI visited target areas that had the highest concentration of SCC according to a city-wide census conducted by CINI in 2019. They collaborated with SCC and young people who were members of children’s groups or local governance platforms to jointly identify potential peer leaders. The children were eventually democratically selected by members of the children’s groups, based on their ability to act as representatives of SCC.

A series of capacity building trainings have been organized to inculcate new skills among the Street Champions. Inputs have been given on a wide range of topics which include Child Rights, UNCRC, Research concepts and Tools, leadership and communication, with the help of a training kit on Participatory Vulnerability Assessment and Service Mapping.

The Street Champions then gathered quantitative as well as qualitative evidence from 450 of their peers and participated in interviews conducted by CINI with 22 service providers and duty bearers from the Government relevant to children’s health, protection, and development. The study therefore provided unique, child-led evidence on the status of SCC’s rights in the city of Kolkata and the specific gaps in services and support for this group. Due to the onset of the COVID-19 pandemic and related lockdown restrictions in 2020, this study also provided insight into the impact of the coronavirus crisis on SCC’s ability to enjoy their rights (4).

### 2.2 Tools of evidence generation

Tools for evidence collection used in this study included a suite of methods to generate data sets which provide information about complex vulnerabilities faced by SCC, such as:

- Systematic review and analysis of legislation and policies
- Rapid Situation Assessment Surveys
- Focus Group Discussions
- Semi-structured interviews

Research tools were constructed based on a framework of enquiry following draft indicators of child rights and the UNCRC General Measures of Implementation. Street Champions were facilitated to participate in the development of research tools and approaches. They prioritized 10 Articles of UNCRC and targets specified in UNGC 21 on which tools for data collection were developed. They took part in forming questions and translating them into languages that would make it easier to communicate with their peers. After the development of the tools, Street Champions took part in field testing of the tools and suggested modifications to ensure proper understanding of the questions by their peers.

### 2.3 Analytic techniques

Both quantitative and qualitative techniques were applied to analyze the findings generated through the different methods. The findings were then analyzed together to draw and support a set of key claims to SCC’s complex vulnerabilities as well as access and availability of services. The findings were then reviewed with Street Champions and they were discussed in detail. Following the review, workshops were held with the Street Champions where they drafted their recommendations based on the research findings, along with the executive summary of the research. They were then translated into English. Responses to the Rapid Situation Assessment were analyzed by calculating the percentage of responses to each question, which were structured and dichotomous or with multiple choices. The responses were codified at first, after which the calculation of percentage was conducted. The focus group discussions and interviews were analyzed using Grounded Thematic Analysis, following a combination of closed and open coding to identify specific themes in the data sets about protection, participation and provision rights. The grounded- theory approach was selected for the data-analysis stage of a study because this process involves the critical review of responses to determine appropriate coding and the formation of themes from those codes. Researchers can conduct thematic analyzes on the transcriptions of participants’ responses to interview questions, other dialogue, or responses to open-ended questions (5).

The project could ensure the participation of Street Champions in this stage. They were consulted during the formation of themes and they explained about their observations for thematic analysis. The findings generated through the analysis of quantitative and qualitative data were collated for the preparation of the final report of this participatory research. For the dissemination of the findings with the SCC and the community, the project team developed different child- friendly communication materials (flip book, animation, storybook etc.). Street Champions were explained about these materials and how they could use them for dissemination of the research findings.

## 3 Results

The centrality of participation maintained throughout the research revealed the experienced realities of SCC, thereby contributing toward understanding the context from the lens of children themselves (6). Without such understanding, interventions have little local relevance and meaningful impact and can cause actual harm to the target populations, creating worse outcomes than if there had been no intervention [(7); Rudnick et al., 2019].

Findings on health and well-being, protection and participation of SCC served as evidence, which the Street Champions shared with local and international stakeholders, to demand inclusion in policies and programs. With training and support, Street Champions were able to make themselves heard and initiate change in their communities as well as act toward ensuring their inclusion in local governance. Some of the key takeaways from the research are as follows:

### 3.1 Health and well-being

→ Living and working on the street presents challenges to the physical and mental health of street- connected children.
→ Due to high mobility, SCC are often invisible to healthcare service providers and policymakers.
→ Healthcare is free and universal, yet poor education, lack of legal identity and negative perceptions serve as barriers to the uptake of services among SCC.
→ SCC are likely to develop chronic conditions and other communicable diseases which they ignore and lack the knowledge and courage to access healthcare facilities.
→ Together with a lack of health education, poor living conditions expose children to risks regarding sexual and reproductive health. These include sexually transmitted diseases and unwanted teenage pregnancies.
→ Girls face additional challenges in terms of their access to reproductive and menstrual health due to poor sanitation and increased exposure to sexual violence.
→ SCC may resort to substance misuse either as a coping strategy, in response to handling peer pressure or as a result of family patterns of behavior.
→ Risky behaviors such as early involvement in sexual activities, or substance misuse further put SCC’s emotional, mental, and physical health at stake.

### 3.2 Social determinants of health including safety

→ Homelessness is one of the worst conditions of existence for a person deprived of security, growth and dignity of life.
→ SCC and their mothers have multiple experiences of violence in different spheres of their lives, from within their own families to workspaces.
→ While there are existing support mechanisms to protect children from abuse and violence, SCC are not well-informed about reporting mechanisms and often possess a fear toward the police. Violence is widely underreported by SCC, and lack of information as well as mistrust toward authorities play a major role in children’s access to adequate protection. In fact, the one of the recommendations from the SCC developed on the basis of research was *“We need the police to understand us, be informed and sensitive towards the vulnerabilities that we face and discuss with us the ways in which reaching out to* them and lodging complaints could be child-friendlier so that those of us who cannot read or write can also understand the processes and go forward with it any time that they need to, without being scared.”
→ SCC acquire protection and support from the street-connected community and peer groups, urban local bodies and NGOs.
→ Street-connected children’s presence in public spaces is not well tolerated by many, who perceive them as being troublemakers and a nuisance to the community. The study suggests that not only the general public, but also service providers in many cases hold negative attitudes toward street-connected children. Such views are influenced by the perception of the street-connected population as one that unlawfully ‘occupies’ or ‘trespasses’ on spaces that do not belong to them. As a result, children are disproportionately vulnerable to harassment, verbal and physical violence, mistreatment by community members, including those who may take advantage of their presence on the streets, such as business owners who engage street-connected children with occasional work. This increases children’s feeling of being unwanted and excluded, as well as their mistrust in the adults surrounding them.
→ As a result of the abusive treatment by adults in society, SCCs are distrustful of adult intervention in their lives: the research shows that almost half of the SCCs surveyed feel that they do not have anyone in their lives whom they can trust or talk to, even if they get hurt.

### 3.3 Participation and inclusion

- The social misconceptions around SCC as being criminals, problematic and a nuisance to public order often lead to their criminalization or victimization (8).
- There are no programs targeted at reframing the social attitudes toward street- connected children or to counter discrimination against them.
- SCC are not encouraged to participate in public decision-making and governance platforms that exist to engage communities and children in issues affecting their lives.
- SCC are not fully included in official population data and statistics, posing barriers to inclusive planning of services and policies (9).
- A lack of cross-sector coordination exists consistently between different government departments, regarding specifically addressing the vulnerabilities of SCC.

Based on the findings, Street Champions developed recommendations for service providers as well as NGOs.

### 3.4 Building resilience through participatory, meaningful negotiation and dialogue

To engage SCC in meaningful dialogs and negotiations with duty-bearers, it is crucial to create spaces for them that are safe and appropriate for them to do so. Effective and meaningful dialogs between the SCC and duty-bearers can bring about an increased understanding of those in authority about the children as valid participants in the decision- making processes, rather than victims or criminals. Street Champions, with the evidence that they collected through the research, were able to make themselves heard and initiate change in their communities. A scope for possible space for dialogue was identified to be with the City Police, who play a significant role in identifying the vulnerabilities of children and protecting their rights. Children living on the streets often possess fear and resistance during interaction with police officials. This creates a barrier between the services delivered by the Police and placing complaints by street- connected children. This issue was identified by Street Champions through the research. To break the barrier and to develop a positive relationship between the police and the SCC, a friendly visit by Street Champions and their street-connected peers to the local Police Stations was organized by CINI. Before visiting the Police Station, a preparatory workshop was organized to discuss with children the issues that they face in their area and to know what kind of help they want from the Police. During the visit, Street Champions and their peers interacted with the Police Officers presented the prioritized issues and discussed the possible solutions. Their vulnerabilities were acknowledged by the police, and this visit followed by many others led to a more effective reporting and response mechanism between the children and the police. In fact, as soon as the news of the pandemic was circulated within the city, police officials went around informing pavement dwellers and children that a lockdown would be implemented soon, and advised them to return to their native places, if possible. In the form of distribution of cooked food from community kitchens, the families living on the streets as well as slums of the hardest hit locations have been reached by the police. Besides food relief, female police officials also reached out with the distribution of Sanitary Napkins to girls living on the streets. Apart from preparing, informing and reassuring the community in light of the lockdown announced on the onset of pandemic, the street champions have been helping the local government support the most vulnerable families by identifying several issues regarding access to food, medical services and social care within their community and the categories at highest risk, such as pregnant women and girls and people unable to access food.

While the vaccination against COVID-19 for the age group 15–18 years opened up from 3rd January 2022 across India, an identification (ID) document was made mandatory for the access to the vaccine. As a member of the District Level Immunization Task Force, CINI had been highlighting the issue of equitable vaccination across the district of Kolkata where there is a significant portion of children living on the streets, unregistered slums, children rescued from trafficking; who are devoid of any Identity document. Street Champions, in their own localities, identified their peers within the eligible age group who did not have any documents and reached out to CINI as well as the local and district level service providers for ensuring their vaccination. Within the city-level network of NGOs working with SCC in Kolkata, the issue regarding the issue of legal identity that created a barrier in accessing vaccination was discussed. After long discussions with authorities who create and process legal identity documents as well as National and District Government Healthcare providers, a joint exception mechanism was developed to ensure delivery of benefits and services to the children in the absence of identification (ID) documents. In this mechanism, more than 10 children could get vaccines by registering with the legal identity document of one government service provider. After the mechanism was launched, quasi- judicial bodies and government bodies such as the State Child Rights Commission, Kolkata Municipal Corporation, Directorate of Child Rights and Trafficking started showing interest to collaborate with CINI to ensure vaccine protection for children in the streets and slums of Kolkata. A special vaccination camp was started, in collaboration with Kolkata Municipal Corporation and the West Bengal Commission for Protection of Child Rights. Street Champions mobilized more than 500 of their peers who did not have any identification documents, to ensure their vaccination, counseled them to reduce their hesitancy against uptake of vaccines, and also assisted them to the vaccination centers.

The resilience of Street Champions especially during the pandemic was shared as examples of young people’s agency on many platforms such as the Consortium for Street Children’s report on Covid-19 and SCC: impacts, responses, and Opportunities published in 2021, written by Ruth Edmonds and Shona Macleod.

The Street Champions’ role as agents of change was also mentioned in “Children as Agents of Positive Change” published by the Office of the Special Representative of the Secretary-General on Violence against Children in the year 2021 to provide an overview of the different actions taken forward by children mostly in times of COVID-19, but not limited to it.

The Street Champions, after gaining insights into their vulnerabilities, raised those issues and shared their actions of change on local, national and global platforms to bring about changes (10). Some of the crucial dialogs and negotiations among Street Champions and duty-bearers are mentioned below:

1. On June 2023, Street Champions participated in the development of General Comment No. 26, on Children’s Rights and the Environment with a Special Focus on Climate Change.
2. On 15th March 2023, Street Champions participated in a children’s consultation on inclusive social protection organized by the United Nations Human Rights Office. This consultation was an opportunity for the children to contribute to the preparation of the 2023 Report to the Human Rights Council on Inclusive Social Protection, prepared by the UN High Commissioner for Human Rights. The children suggested solutions to address the issues faced by them in terms of the difficulties they faced due to experiences of exclusion, which they identified through the research.
3. On 25th October 2022, Street Champions received a letter of appreciation from Dr. Najat, the United Nations Special Representative of the Secretary-General on Violence against Children. This letter was in response to a letter previously submitted to her by the Street Champions, in which they described the impact of climate change in their lives, for an advocacy brief that would be launched by the UN during the General Assembly. The response from the street champions was greatly appreciated and encouraged by the UN, as visible in the letter sent by Dr. Najat which can be viewed here: https://drive.google.com/file/d/13Kwg9iekpxgtBwjtQUENGETe5ljQMxrW/view?usp=sharing.
4. On 1st October 2022, Street Champions raised recorded questions to Najat Malla M’jid, the United Nations Special Representative of the Secretary-General on Violence against Children (SRSG VAC). The questions to the world leaders and the United Nations were played on 1st of October 2022, when Dr. Najat briefed children on the margins of the UN General Assembly 77, on her report to the General Assembly. Along with videos, children also submitted a letter to the UN, which was published by the SRSG VAC in October 2022, in her report titled: The Climate Crisis and Violence Against Children. The letter sent to the UN by the SCC affiliated with CINI can be viewed on page 13 of the report available at the following link: https://violenceagainstchildren.un.org/news/the-climate-crisis-and-violence-against-children.
5. On 5th April 2022, Street Champions shared their views with the United Nations Committee on Economic, Social and Cultural Rights to contribute toward drafting a General Comment on Sustainable Development and Economic, Social and Cultural Rights. India was greatly appreciated by the Committee for making valuable contributions to the General Comment about Sign language and universal access to the Government’s schemes and services.
6. In March 2022, Street Champions participated in an online consultation with the SRSG VAC where she shared her report to the Human Rights Council and answered questions made by children. One of them asked the SRSG about how SCC can participate in reviewing States’ reports on VAC to the United Nations. The recorded version of the online event is available at this link: https://www.facebook.com/UNviolenceagainstchildren/videos/477988740647699/ and his question is after 45.30 min of the video.
7. On 1st March 2022, one of the Street Champions addressed the 46th Human Rights Council of the United Nations A link from the channel United Nations (UNTV) where the address was broadcasted is available here (Her speech is in the time stamp 55 min 35 s): https://media.un.org/en/asset/k1k/k1k78p7c5l (11).
8. In 2021, the diverse roles played by CINI in facilitating SCC to prevent, address and report VAC instances were documented in the publication by United Nations SRSG/VAC named “Children as Agents of Positive Change: A Mapping of Children’s Initiatives Across Regions, Toward an Inclusive and Healthy World Free From Violence,” p. 26–27: https://rm.coe.int/unsrsg-2021-children-as-agents-of-positive-change/1680a2366f.
9. The submission made by SCC that CINI works with, on mental health to the high-level political forum organized by OHCHR was shared on the social media platform on August 2021 by the United Nations Office on Violence Against Children: https://www.facebook.com/UNviolenceagainstchildren/videos/621369498833154/?extid=WA-UNK-UNK-UNK-AN_GK0T-GK1.
10. In between lockdowns, CINI mobilized its network with local NGOs as well as children from different parts of the city and arranged a webinar with the State Commission for Protection of Child Rights as well as district and state level duty bearers from health, education, and protection departments. The webinar was arranged by facilitating network partners, children nominated from other NGOs as well as Street Champions to come together and share the vulnerabilities faced by children requiring immediate responses, especially during the pandemic. Street Champions raised issues they identified through research, which included increased experiences of abuse, and lack of access to shelter and education support. Some immediate responses such as resuming activities at open shelters for SCC, and initiating remote learning through radio were observed to be launched by the district and State Government officials immediately after those webinars.

## 4 Conclusion

The United Nations Adolescent Well-being Framework includes domains for connectedness (12), positive contribution to society, and agency and resilience – that is, being ‘empowered to make meaningful choices and to influence their social, political, and material environment’ (Ross et al., 2020). However, investments in young people adhering to the SDGs remain largely confined to health, education and protection from violence, with little attention to adolescent voice, agency, civic engagement and political participation (Sheehan et al., 2017; Guglielmi et al., 2022).

It is important that all children and young people can learn to participate in programs which directly affect their lives. This is especially for disadvantaged children who can act together to reduce discrimination and repression and speak about equal rights through dialogs with adults. In this participatory research, children and young people meaningfully participated in each of the steps and collaborated with adults. Children and young people were involved in participatory research as researchers rather than merely subjects of research (13). This project has helped Street Champions (representatives of SCC) acquire skills for effective communication and meaningful dialogs with the duty bearers.

## Data availability statement

The original contributions presented in the study are included in the article/supplementary material, further inquiries can be directed to the corresponding author.

## Ethics statement

The studies involving humans were approved by CINI Institutional Ethics Committee. The studies were conducted in accordance with the local legislation and institutional requirements. Written informed consent for participation in this study was provided by the participants’ legal guardians/next of kin.

## Author contributions

DB: Writing – original draft. SC: Writing – original draft.

## References

[1] Before Not After. (2020). Evaluation of CINI’s preventative approach to child protection in India. Available at: https://cdn1.sph.harvard.edu/wp-content/uploads/sites/2464/2020/01/BeforeNotAfterII-FINAL.pdf

[2] Wellcome Trust, (2019). Empowering street children to inform responses to their health needs. Available at: https://wellcome.org/grant-funding/people-and-projects/grants-awarded/empowering-street-children-inform-responses-their

[3] Convention on the Rights of the Child (2017). General comment No. 21 on children in street situations. Available at: https://www.streetchildren.org/wp-content/uploads/gravity_forms/1-07fc61ac163e50acc82d83eee9ebb5c2/2017/07/General-Comment-No.-21-2017-on-children-%20in-street-situations.pdf

[4] Heydarian NM. Developing theory with the grounded-theory approach and thematic analysis. Washington, D.C.: Association for Psychological Science (2016).

[5] Edmonds R. Making children’s ‘agency’ visible: towards the localisation of a concept in theory and practice. Glob Stud Child. (2019) 9:200–11. doi: 10.1177/2043610619860994

[6] Presler-Marshall E Oakley E Jones N Baird S Alheiwidi S Małachowska A. How do gender norms shape adolescent trajectories in post-pandemic Jordan? London: Gender and Adolescence: Global Evidence (2023).

[7] StreetInvest, (2021). Our work in India, street champions lead the way in Kolkata. Available at: https://streetinvest.org/india/

[8] CINI. (2021). Kolkata vulnerability and service mapping: briefing papers. Available at: https://www.streetchildren.org/resources/kolkata-vulnerability-and-service-mapping-briefing-papers/

[9] CINI, (2023). Response to the questionnaire on decriminalization of homelessness and extreme poverty to inform the special rapporteur on the right to adequate housing and the special rapporteur on extreme poverty and human. Available at: https://www.ohchr.org/en/calls-for-input/2023/call-input-decriminalization-homelessness-and-extreme-poverty

[10] Breakthrough, (2018). Street children – statistics, their lives and why we have to care. Available at: https://inbreakthrough.org/street-children-statistics-lives/

[11] Dynamo International. (2021). Children share their views with the human rights council and more! Available at: https://dynamointernational.org/en/children-share-their-views-with-the-human-rights-council-and-more/

[12] Spyrou S. The limits of children’s voices: from authenticity to critical, reflexive representation. Childhood. (2011) 18:151–65. doi: 10.1177/0907568210387834

[13] The Lancet Child Adolescent Health. Advancing the rights of street and working. Lancet Child Adolesc Health. (2023) 7:223. doi: 10.1016/S2352-4642(23)00058-5 36948659

